# Our Insurance Act Department

**Published:** 1912-04-20

**Authors:** 


### Our Insurance Act Department.

### ANSWERS TO CORRESPONDENTS.

### Payments to Medical Men under the Act.

"Medicus" writes: On July 15 presumably patients will choose a doctor under the Act. When will the first payment be made to medical men? Medical benefit not coming in until January 15, 1913, will the doctors' fees accrue only from that date?

*** This is one of those questions of "detail" which have still to be settled in regulations made by the Commissioners. It is clear from Section 15 (1) of the Act that nothing can be done until the formation of Insurance Committees, and that these committees can only make arrangements with duly qualified medical practitioners in accordance with regulations made by the Insurance Commissioners. These regulations are to provide for the preparation and publication of lists of medical practitioners who have consented to act; and if the list so prepared is unsatisfactory, the committee may be authorised to make any other arrangement. Upon any view of the matter it is not likely that payments to medical practitioners will begin before insured persons become entitled to benefits.

### Nurses and Insurance under the Act.

A Nurse writes: Will you kindly let me know (1) whether a nurse or matron (who is in perfect health) will be compelled to come under the Insurance Act; (2) whether a birth certificate will be required?

*** (1) The National Insurance Act, 1911, applies to employment in the United Kingdom "under any contract of service . . . written or oral, whether expressed or implied, and whether the employed person is paid by the employer or some other person . . . and whether paid by time or by the piece or . . . without any money payment." This comprehensive definition of the employments to which the Act applies would clearly extend to the occupation of a matron or a nurse, but it may be that these persons would come within the exceptions. Thus the Act does not apply to (*a*) "employment of a casual nature otherwise than for the purposes of the employer's trade or business"; (*b*) employment otherwise than by way of manual labour and at a rate of remuneration exceeding in value £160 a year, or in cases where such employment involves part-time service only at a rate of remuneration which, in the opinion of the Insurance Commissioners, is equivalent to a rate of remuneration exceeding £160 a year for whole-time service." Finally, a person in receipt of a pension or annuity of £26 or upwards not dependent upon her personal exertions, or who is ordinarily and mainly dependent for her livelihood upon some other person, is entitled to a certificate of exemption. Unless the classes of persons mentioned by "Nurse" can be brought within these exceptions it seems to be clear that they are entitled to the benefits of the Act. (2) With regard to the production of birth certificates, the question when and in what circumstances they are to be forthcoming will have to be settled by regulations which are not yet issued.

